# Effects of Angiopoietin-2 on Transplanted Mouse Ovarian Tissue

**DOI:** 10.1371/journal.pone.0166782

**Published:** 2016-11-21

**Authors:** Hye Won Youm, Jaewang Lee, Eun Jung Kim, Hyun Sun Kong, Jung Ryeol Lee, Chang Suk Suh, Seok Hyun Kim

**Affiliations:** 1 Department of Obstetrics and Gynecology, Seoul National University Bundang Hospital, 82 Gumi-ro 173 Beon-gil, Bundang-gu, Seongnam-si, Gyeonggi-do, 463-707, Korea; 2 Department of Obstetrics and Gynecology, Seoul National University College of Medicine, 101 Daehak-ro, Jongno-gu, Seoul, 110-744, Korea; West China Second Hospital, Sichuan University, CHINA

## Abstract

Transplantation of ovarian tissue (OT) is currently the only clinical option to restore fertility with cryopreserved OT. However, follicle loss caused by ischemia and slow revascularization occurs in transplanted OT. To shorten the ischemic period and promote angiogenesis, some angiogenic factors have been used. Angiopoietin-2 (Ang2) is one of the major angiogenic factors and has been reported to promote blood vessels and increase vascular permeability in ischemic and/or hypoxic environment. This study was performed to investigate the effects of Ang2 on follicle integrity and revascularization of transplanted mouse OT. Five-week-old B6D2F1 female mice were divided into a control group and two Ang2 groups, followed by ovary collection and vitrification. After warming, the ovaries were autotransplanted into kidney capsules with/without Ang2 injection (50 or 500 ng/kg), and then the mice were sacrificed at days 2, 7, 21, and 42 after transplantation. A total 2,437 follicles in OT grafts were assessed for follicular density, integrity, and classification by using hematoxylin and eosin staining. Apoptosis and revascularization were evaluated by using TUNEL assay and CD31 immunohistochemistry, respectively. Serum follicle-stimulating hormone (FSH) levels were measured by using enzyme-linked immunosorbent assay. Both Ang2 groups showed remarkable increase in morphologically intact follicle ratio across all grafting durations except D21. The numbers of CD31(+) vessels were significantly increased in both Ang2 groups compared with the control group at all durations, except in the 50 ng Ang2 group at D42. However, the mean numbers of follicles of the grafts, apoptosis ratios, and serum FSH levels showed no significant differences among the groups. Our results show that Ang2 treatment significantly increased the intact follicle ratios and the number of blood vessels of the mouse OT grafts. However, further studies performed with large animal or human OT are necessary before clinical application for fertility preservation in cancer patients, and the reliability of the systemic effects of Ang2 should be verified.

## Introduction

Cancer treatment has had remarkable progress, which has enabled cancer patients to survive. However, some female survivors suffer from infertility caused by chemotherapy and/or radiotherapy, which lowers their quality of life [1,2]. To overcome these problems, several fertility preservation options have been introduced including oocyte, embryo, and ovarian tissue (OT) cryopreservation. Ovarian tissue cryopreservation followed by transplantation has been considered as an experimental procedure; however, > 60 children have been born as a result of this technique [3]. Therefore, OT cryopreservation and transplantation are regarded as an option that should be offered to female cancer patients. This procedure has many advantages for women who do not have a spouse and/or no time to delay chemotherapy. Moreover, for prepubertal female patients, it could be the only option for fertility preservation [4,5].

After OT cryopreservation and transplantation, large amounts of ovarian follicles are damaged and reduced by cryo-damage and ischemic injuries. Previous reports indicated that ischemic injuries during the transplantation process were more detrimental to OT grafts than cryo-damage [6,7]. Imbalance between blood demand and blood supply caused by impaired and insufficient vascularization in the transplanted OT results in a severe hypoxic condition and cell death [8]. To reduce ischemic damage by shortening the ischemic period, angiogenesis should be facilitated.

Angiogenesis is the process of new vessel formation from the preexisting blood vasculature. It is essential for several physiological mechanisms including embryogenesis, wound healing, and malignancy expansion [9]. Accumulating data have shown that, among the many angiogenic factors, the vascular endothelial growth factor (VEGF) plays an important role in the induction of vascular permeability and neovascularization [10,11]. Recently, the mechanism of angiogenesis has focused on the growth factor angiopoietin (Ang), which was discovered in 1996 [12] and defined to regulate the formation of new blood vessels with VEGF and modulate inflammatory processes [13]. Angiopoietin-1 (Ang1) and angiopoietin-2 (Ang2) have been proven to be directly involved in blood vessel formation. Ang1 plays a role in maturation and stabilization of new blood vessels, inhibition of endothelial apoptosis, and reduction of vascular permeability in the stable condition without hypoxia [14,15]. Ang2, an antagonist of Ang1, is expressed in sites of blood vessel remodeling and has been reported to promote blood vessels plasticity, destabilization, and permeability—especially in ischemic and hypoxic environments such as transplanted or injured tissues [13,16–18]. Furthermore, several studies found that Ang1 and Ang2 were expressed in ovarian follicles and have a regulatory role in ovarian angiogenesis [19,20]. However, the exact mechanisms of their functions have not been well proven yet.

Thus, we theorized that Ang2 would have positive effects on the survival of OT grafts that suffer from ischemic injuries during transplantation and reperfusion processes. We performed this study to improve the follicle survival, integrity, and revascularization of OT grafts through Ang2 treatment, and to assess the effect of Ang2 on transplanted mouse OT.

## Materials and Methods

### Experimental Animals

Five-week-old B6D2F1 female mice (Orientbio, Seongnam, South Korea) were housed under a 12-hour light/dark cycle and fed ad libitum. The experimental protocols and animal handling procedures were performed with the approval of the Institutional Animal Care and Use Committee of Seoul National University Bundang Hospital.

### Vitrification of Mouse Ovaries

The mice were anesthetized with intraperitoneal (IP) injection of 30 mg/kg zolazepam + tiletamine (Zoletil; Virbac, France) and 10 mg/kg Xylazine (Rompun; Bayer, Germany) followed by ovary extraction. Then the ovaries were vitrified by the two-step method described in our previous report [21] as follows: the OTs were placed in the equilibration solution consisting of Dulbecco’s phosphate buffered saline (D-PBS; Gibco; Paisley, UK) supplemented with 20% fetal bovine serum (FBS; Gibco), 7.5% ethylene glycol (EG; Sigma; St. Louis, MO), and 7.5% dimethylsulfoxide (DMSO; Sigma) for 10 minutes at room temperature (RT) and moved to the vitrification solution composed of D-PBS containing 20% FBS, 20% EG, 20% DMSO, and 0.5 M sucrose (Sigma) for 5 minutes at RT. The ovaries were placed on an electron microscopic (EM) copper grid (JEOL; Tokyo, Japan) and the droplet of vitrification solution on the grid was removed by using underlying sterilized filter paper. We placed the EM grid directly into liquid nitrogen (LN2) and then put the grid into a 1.5 mL of cryovial (Nunc; Roskilde, Denmark) already set in a cane and located in LN2.

### Warming and Autotransplantation

Vitrified OTs were warmed through stepwise dilution of sucrose at concentrations of 1.0, 0.5, 0.25, and 0 M in D-PBS medium supplemented with 20% FBS. Then autotransplantation was performed as described in our previous report [22]. After analgesia of the mice, a small hole was made on their kidney capsules by using fine forceps and the vitrified-warmed ovaries were inserted under both of the kidney capsules.

### Angiopoietin-2 Injection

The mice (n = 175) were randomly divided into three groups according to Ang2 treatment (0 ng/kg Ang2, control: n = 56; 50 ng/kg Ang2: n = 60; and 500 ng/kg Ang2: n = 59). The mice were given IP injections with 50 or 500 ng/kg Ang2 (R&D System; Minneapolis, MN) at 18 hours and 30 minutes before the transplantation procedures because the half-life of Ang2 was 18 hours [23]. The first Ang2 injection (before 18 hours) was administered to act on the OT grafts after transplantation; and the second injection (before 30 minutes), to prolong the Ang2 effect during neovascularization. For the control group, normal saline was also injected intraperitoneally by using the same scheme described earlier.

### Gross Evaluation of Ovarian Graft Vascularization

The ovaries from another 25 mice were autotransplanted into the subcutaneous pockets after ovariectomy without vitrification and warming. Among the 25 mice, 12 belonged to the control group, and 13 were treated with 500 ng/kg Ang2. Seven days after autotransplantation, the gross morphology and vascularity of the OT grafts were evaluated. When precise blood vessel branches were found between the OT grafts and the recipient’s body, and the colors of grafts ranged from yellow to red, the OT grafts were regarded as well vascularized; whereas white OT grafts with rare or no blood vessels and follicles were considered as poorly vascularized.

### Recovery of Ovarian Grafts and Histological Staining

The autotransplanted OT grafts were collected at days (D) 2, 7, 21, and 42 after transplantation, respectively, and fixed in 4% paraformaldehyde. The fixed ovaries were embedded in paraffin blocks and serially sectioned to with 5 μm thickness. The sections on the slides were stained with hematoxylin and eosin (H&E) solution (Merck; Darmstadt, Germany) for histological evaluation. The other sections were used for immunohistochemistry (IHC) and TUNEL assay (Roche; Mannheim, Germany). The subcutaneously transplanted OT grafts from the 25 mice were collected at D7 after transplantation and observed for the appearance of surrounding vessels.

### Follicle Classification and Morphological Analysis

Ovarian follicles were observed under a light microscope (Nikon; Tokyo, Japan) at x400 magnification and classified into four developmental stages according to the following categories established by Lundy et al. [24]: primordial, primary, secondary, and antral follicles.

1. Primordial: single layer of flattened pregranulosa cells
2. Primary: single layer of granulosa cells, one or more of which being cuboidal
3. Secondary: two or more layers of cuboidal granulosa cells, with the antrum absent
4. Antral: multiple layers of cuboidal granulosa cells, with the antrum present

For morphological evaluation of follicle integrity, the following criteria were used to assess the follicles as grade (G) 1, 2, or 3 according to three states [21,25] as follows.

G1: good, intact follicle and oocyte
G2: fair, granulosa cells pulled away from the edge of follicles, but with intact oocyte
G3: poor, disruption, loss of granulosa and theca cells, and broken or missing oocyte

To avoid miscounting, the OT follicles, when they contained oocytes, they were analyzed in only one section per ovary.

### Measurement of Follicle-Stimulating Hormone Level

Mouse blood samples were collected through cardiac punctures at the time of OT graft collection. From whole blood, serum was separated through centrifugation. Serum follicle-stimulating hormone (FSH) levels were measured by using an enzyme-linked immunosorbent (ELISA) assay kit (Endocrine Technologies; Newark, NJ) according to manufacturer instructions. The intra- and inter-assay coefficients of variation were 6.35% and 5.88%, respectively, with a sensitivity of 0.5 ng/mL.

### Analysis of Apoptosis

The apoptosis of the OT follicles was analyzed by using TUNEL assay. In brief, after deparaffinization, rehydration, and treatment of 0.1% Triton X-100 (Amresco; Cleveland, OH) in citrate buffer, the slides were incubated with TUNEL reaction mixture for 1 hour at 37°C in a humidified dark chamber, followed by washing with D-PBS. Then, the slides were mounted with VECTASHIELD mounting medium with 4',6-diamidino-2-phenylindole (DAPI; Vector Laboratories Inc.; Burlingame, CA), and examined under an inverted Zeiss AX10 microscope (Carl Zeiss AG; Oberkochen, Germany).

TUNEL-positive cells produced green fluorescence at excitation wavelengths ranging from 450 to 500 nm and detection capabilities ranging from 515 to 565 nm. When 30% of the cells in one follicle were TUNEL positive, the follicle was regarded as apoptotic. DAPI reached excitation at about 360 nm and emitted at 460 nm when bound to DNA, producing blue fluorescence.

### Immunohistochemistry for CD31

The intensity of the blood vessels was investigated by IHC with anti-CD31 antibody. Among the serially sectioned OT slides, we chose one of the largest sections (middle part of OT graft). The section that had both periphery and center of the OT was used for vascularity analysis as a representative section. For reagents used in this procedure that were purchased from Dako in Denmark, we did not indicate the company. The unstained paraffin slides were deparaffinized and rehydrated. Then, the target retrieval solution (pH 9.0) was treated for antigenic retrieval. The sections were treated with peroxidase-blocking solution followed by incubation with an anti-C31 antibody (1:200 dilution; Abcam; Cambridge, UK) for 1 hour. After washing, the sections were treated with EnVision+ HRP and liquid DAB+ substrate followed by counterstaining with hematoxylin. After dehydration, the slides were mounted and examined under the inverted Zeiss AX10 microscope. To calculate vascular density, CD31(+) vessels were counted in three different high power fields (×400) per ovary.

### Statistical Analyses

The result of follicle integrity within the samples was calculated by the chi-square test or analysis of variance (ANOVA) using the SPSS version 12.0 software (SPSS Inc.; Chicago, IL) and GraphPad Prism 5.0 (GraphPad Software; La Jolla, CA). The outcomes were considered statistically significant when the p values were < 0.05.

## Results

### Mean Follicle Numbers

**Fig 1:**
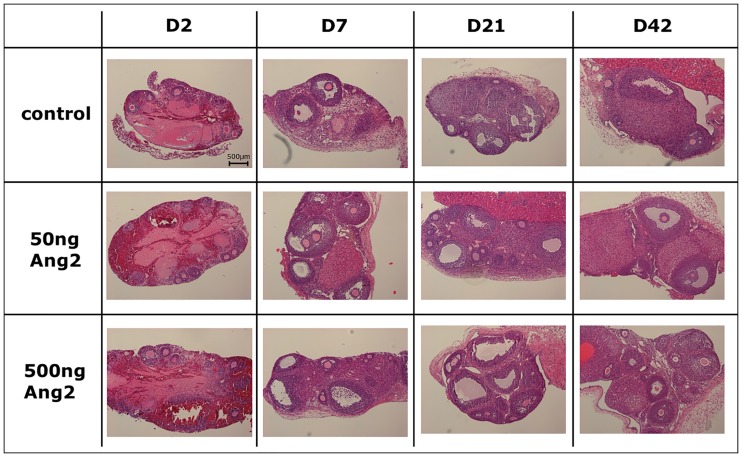
Histology of ovarian grafts (original magnification ×100). The horizontal axis represents the days after OT transplantation. The vertical axis represents the control and Ang2-treated groups (control, no treatment of Ang2; 50 ng Ang2, injection of 50 ng/kg Ang2; 500 ng Ang2, injection of 500 ng/kg Ang2; D2, D7, D21 and D42, 2, 7, 21 and 42 days after transplantation, respectively).

A total of 2,437 follicles were morphologically evaluated for follicular density and integrity by performing a histological analysis. The morphology of the OT grafts stained with H&E is shown in Fig 1. On D2, few intact follicles, a large proportion of damaged stromal cells, and plenty of red blood cells were observed in all the groups. The OT grafts were easy to detach from the kidney capsules. However, the morphology of the OTs improved over time post transplantation.

**Fig 2:**
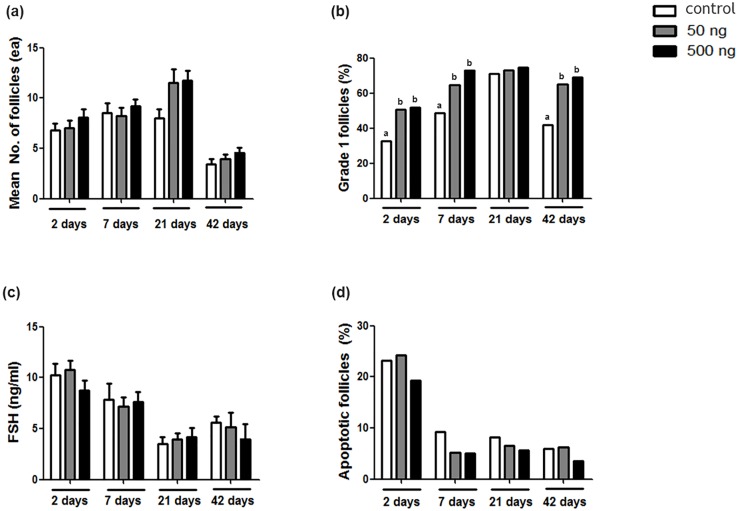
The results of the comparison of (a) the mean numbers of follicles, (b) intact G1 follicle ratios, (c) serum FSH levels, and (d) apoptotic follicle ratios in the OT grafts after Ang2 treatment according to post-transplantation durations. The statistical difference was considered significant when the p value was <0.05 (control, no treatment of Ang2; 50 ng Ang2, injection of 50 ng/kg Ang2; 500 ng Ang2, injection of 500 ng/kg Ang2). Different superscript letters indicate statistically significant differences (p < 0.05), and the superscripts were used for each group of the same transplantation duration separately.

Fig 2(a) represents the follicular density of the three groups according to post-transplantation duration. The mean numbers of follicles were relatively higher in the Ang2-treated groups in all the durations but did not significantly differ among the control and two experimental Ang2-treated groups at D2 (control: 6.8 ± 0.7; 50 ng Ang2: 7.0 ± 0.8; and 500 ng Ang2: 8.1 ± 0.9), D7 (control: 8.5 ± 1.0; 50 ng Ang2: 8.2 ± 0.8; and 500 ng Ang2: 9.2 ± 0.7), D21 (control: 8.0 ± 0.8; 50 ng Ang2: 11.5 ± 1.3; and 500 ng Ang2: 11.8 ± 1.0), and D42 (control: 3.4 ± 0.5; 50 ng Ang2: 3.9 ± 0.5; and 500 ng Ang2: 4.6 ± 0.5) post transplantation.

### Morphological Analysis of the Grafts

**Fig 3:**
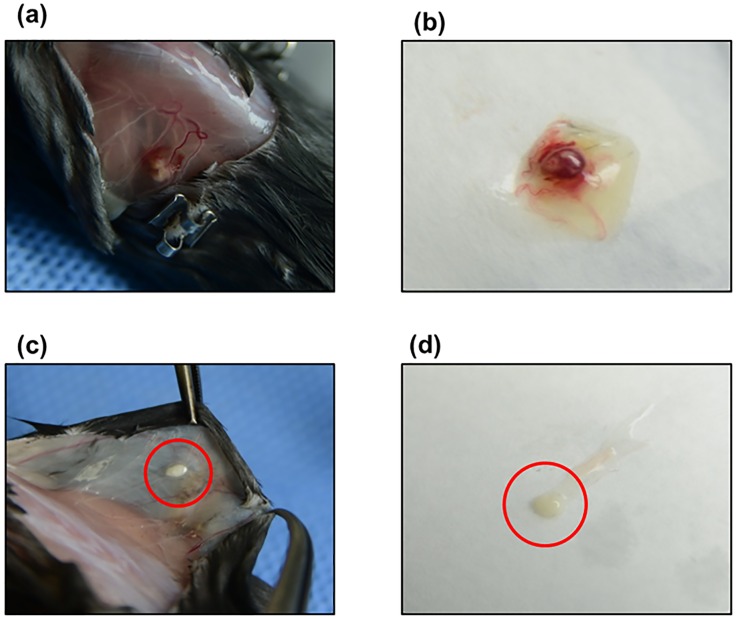
Representative appearance of good and poor vascularization and angiogenesis of OT grafts transplanted into the subcutaneous pockets and recovered on D7 after grafting. (a, b) Good formation of new blood vessels. Several new vessels branching to the OT graft are clearly visible and have a healthy appearance. Three follicles were yellow-white in color in the red OT grafts. (c, d) Poor vascularization. Rare vessels were visible and the OT graft seems to be necrotized and/or degenerated without visible follicles.

Fig 2(b) shows the proportion of the morphologically intact (G1) follicles for the control group and Ang2-treated groups. The G1 follicle ratios were significantly higher in both the 50 and 500 ng groups than in the control group on D2 (control: 32.7%; 50 ng Ang2: 50.8%; and 500 ng Ang2: 51.9%), D7 (control: 48.7%; 50 ng Ang2: 64.5%; and 500 ng Ang2: 73.1%), and D42 (control: 41.7%; 50 ng Ang2: 64.9%; and 500 ng: 69.1%). However, no significant difference in G1 follicle ratio was observed on D21 (control: 71.1%; 50 ng: Ang2 73.2%; and 500 ng Ang2: 74.8%). The overall results of the histological examination, including the mean numbers of follicles and G1 follicle ratios according to the different development stages, are shown in S1 Table. During the OT transplantation periods, some of OTs were degenerated as shown in Fig 3(c) and 3(d), thus we could collect 86.6% (97/112), 88.3% (106/120) and 91.5% (108/118) of OT grafts from the total transplanted OTs of the control, 50 ng Ang2 and 500 ng Ang2 groups, respectively.

### Follicle-stimulating Hormone Level in Serum

To verify the recovery of ovarian function, serum FSH levels were measured for all the groups and durations. As shown in Fig 2(c), the serum FSH levels were high on D2 and decreased thereafter. However, no significant differences were observed among the same duration groups of D2 (control: 10.2 ± 1.2 ng/mL; 50 ng Ang2: 10.8 ± 0.9 ng/mL; and 500 ng Ang2: 8.8 ± 0.9 ng/mL), D7 (control: 7.9 ± 1.6 ng/mL; 50 ng Ang2: 7.2 ± 0.8 ng/mL; and 500 ng Ang2: 7.6 ± 1.0 ng/mL), D21 (control: 3.5 ± 0.7 ng/mL; 50 ng Ang2: 3.9 ± 0.6 ng/mL; and 500 ng Ang2: 4.2 ± 0.9 ng/mL), and D42 (control: 5.6 ± 0.7 ng/mL; 50 ng Ang2: 5.1 ± 1.5 ng/mL; and 500 ng Ang2: 4.0 ± 1.5 ng/mL) post transplantation.

### Apoptosis

A total of 2,991 follicles were evaluated for apoptosis by using TUNEL assay. Fig 2(d) shows the proportion of apoptotic follicles on D2, D7, D21, and D42 after grafting. No significant differences in apoptosis ratio were found among the three groups when the grafts were compared in the same transplantation durations. It is interesting that all the three groups on D2 showed approximately 20% of high apoptotic follicle ratios (control: 23.2%; 50 ng Ang2: 24.3%; and 500 ng Ang2: 19.3%), followed by a decrease to <10% on D7 (control: 9.3%; 50 ng Ang2: 5.2%; and 500 ng Ang2: 5.0%), D21 (control: 8.2%; 50 ng Ang2: 6.6%; and 500 ng Ang2: 5.7%), and D42 (control: 6.0%; 50 ng Ang2: 6.3%; and 500 ng Ang2: 3.6%).

### Blood Vessel Integrity

**Fig 4:**
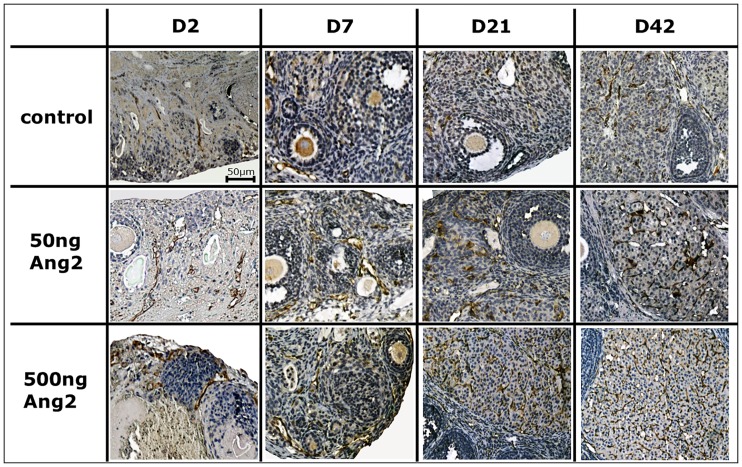
Representative photographs of CD31(+) vessels (dark-brown color) in OT grafts (original magnification ×400). The horizontal axis represents the days after OT transplantation. The vertical axis represents the control and Ang2-treated groups (control, no treatment of Ang2; 50 ng Ang2, injection of 50 ng/kg Ang2; 500 ng Ang2, injection of 500 ng/kg Ang2; D2, D7, D21 and D42, 2, 7, 21 and 42 days after transplantation, respectively).

**Fig 5:**
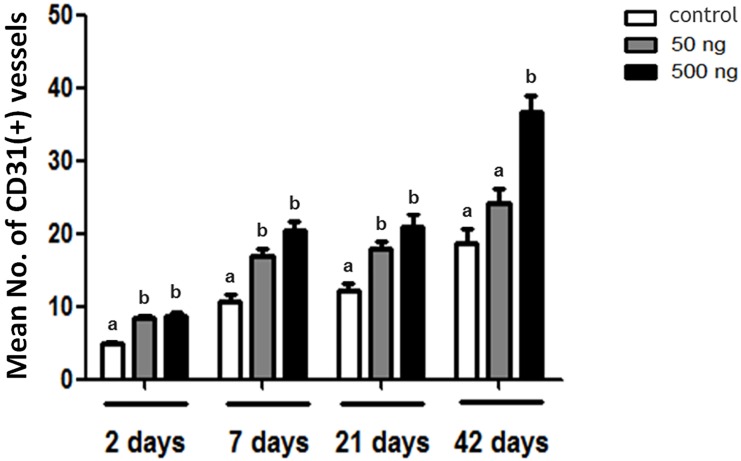
Comparison of the mean number of CD31(+) vessels in the OT grafts after Ang2 treatment according to post-transplantation durations. The statistical difference was considered significant when the p value was <0.05 (control, no treatment of Ang2; 50 ng Ang2, injection of 50 ng/kg Ang2; 500 ng Ang2, injection of 500 ng/kg Ang2). Different superscript letters indicate statistically significant differences (p < 0.05), and the superscripts were used for each group of the same transplantation duration separately.

Fig 4 shows the representative photographs of the OT grafts that were immunohistochemically stained for CD31. The numbers of brown-colored CD31(+) vessels localized in the transplanted OTs were observed on D2, D7, D21, and D42 after grafting. Similar to the outcomes of H&E staining, many impaired stromal cells and ovarian follicles appeared on D2. The numbers of CD31(+) vessels increased with significant differences in both the 50 and 500 ng Ang2 groups, compared with the control group at all the grafting duration of D2 (control: 4.8 ± 0.4; 50 ng Ang2: 8.3 ± 0.5; and 500 ng Ang2: 8.6 ± 0.6), D7 (control: 10.7 ± 1.0; 50 ng Ang2: 16.9 ± 1.0; and 500 ng Ang2: 20.5 ± 1.3), D21 (control: 12.2 ± 0.9; 50 ng Ang2: 17.9 ± 0.9; and 500 ng Ang2: 20.9 ± 1.7), and D42 (control: 18.8 ± 2.0; 50 ng Ang2: 24.2 ± 2.0; and 500 ng Ang2: 36.6 ± 2.2) post transplantation—except in the 50 ng Ang2 group at D42 (Fig 5).

### Gross Evaluation of Vascularization

Fig 3 shows the representative appearance of good and poor vascularization and angiogenesis of the OT grafts transplanted into the subcutaneous pockets and recovered on D7 after grafting. The outcome of gross examination did not significantly differ between the control (good: 72.3% and poor: 27.3%) and 500 ng Ang2 groups (good: 88.5% and poor: 11.5%). However, the tendency of vessel appearance improved in the 500 ng Ang2 group.

## Discussion

In the field of OT cryopreservation and transplantation, the reduction of ischemic damage is one of the most critical issues to improve the survival and integrity of OT grafts, and some angiogenic factors have been considered to up-regulate the neo-angiogenesis of transplanted tissues [26,27]. For the roles of Ang1 and Ang2, several studies reported that Ang1 was related to the embryonic stage of angiogenesis and that Ang2 was associated with post-birth blood vessel remodeling [28,29]. Jeansson et al. demonstrated that in the Ang1 knockout mouse, embryonic blood vessels could not collect the vessel-supporting cells, which resulted in obstruction of angiogenesis [14]. By contrast, in the Ang2 knockout mouse, embryonic angiogenesis proceeded up to a certain level; however, after birth, the postnatal angiogenesis was obstructed [30,31]. In addition, Eltzschig and Eckle showed that Ang2 was expressed 15 times more than Ang1 in the hypoxic environment [32]. When the blood vessels were in the normal and stable environment, Tie-2 receptor in endothelial cells tightly bound to Ang1 of the pericyte. However, in the environment of vessel degradation and/or ischemic hypoxia, which require neovascularization, Ang2 acted as an antagonist of Ang1 and bound to Tie-2 to make the vessel flexible and destabilized. Vessel plasticity should be maintained for remodeling vessels, and then Ang2 induced angiogenesis in the presence of VEGF to make new healthy vessels [32].

In this study, we evaluated the effects of Ang2 on the vitrified-transplanted mouse OT in terms of follicle integrity and vessel formation. All the Ang2-treated groups (50 and 500 ng) showed remarkable increases in morphologically intact follicle ratios across all the grafting durations except on D21. Similar to this result, the numbers of CD31(+) vessels were significantly increased in the Ang2-treated groups when compared with the control group at all grafting durations. Even though no significant difference was found between the 500 ng Ang2 and control groups in the gross examination of OT grafts on D7, we observed that the OT grafts treated with 500 ng Ang2 were better than the control OTs in color, follicle formation, and vessel numbers, which was also proved by the histological examination and CD31 IHC results. These results suggest that the Ang2 treatment increased the angiogenesis of the OT grafts and facilitated the neovascularization, which led to the improvement of follicle survival and integrity. This was similar to the result of Wu et al. in that the treatment of *Salviae miltiorrhizae* via human OT xenografting had positive effects on both blood vessel density and normal follicle ratio, thus showing that increased vascularization could improve follicle integrity as our result [33].

Twenty-one days after transplantation, the OT grafts seemed to be almost completely perfused into the host, repaired their reproductive functions, and were more stable than the OTs on D2, D7, and D42 after transplantation. This is evidenced by the overall results that the follicular densities and G1 ratios were higher on D21 and the FSH levels were lower on D21 than on the other grafting durations. Owing to the upward standardization of the OT grafts on D21, differences between the control and Ang2-treated groups seemed not notable.

The mean follicle numbers were decreased in the control group and both the Ang2-treated groups on D42, which seemed to be due to atrophy of the OT grafts and unstable gonadotropins levels. This phenomenon was similar to human OT transplantation which showed that some OT grafts could not sustain their function over long periods because of follicular loss and stromal destruction due to cryo-damage and cytokine secretion, free radical release, and platelet activation by ischemic injury, causing massive follicle loss and shorter longevity of OT grafts [6,7,34–36]. Despite the overall reduction in follicle density on D42, the positive effect of Ang2 on the G1 follicle ratios and blood vessel densities on D42 was still present, and the morphology of the ovaries represented the healthy conditions of follicles and stromal cells on D42. Ang2 treatment could improve the integrity of follicles and stromal cells with increased angiogenesis, but this improvement could not prevent ovarian follicle loss long after transplantation.

For apoptosis, the proportion of apoptotic follicles was high (almost 20%) on D2, followed by significant decreases on D7, D21, and D42 after grafting. The high apoptosis ratio on D2 could be explained by the fact that the murine OT grafts required 2 days after transplantation for new vessel formation [37]. During the first 2 days of grafting, large numbers of follicles were damaged and degenerated immediately after transplantation due to the lack of active blood vessels. Liu et al. also have reported that follicular apoptosis remarkably increased in the early phase and decreased to normal ranges on day 14 after transplantation [8]. Since rapid recovery and clearance of damaged follicles occurred and only surviving follicles could normally develop from 2 days after transplantation, the high level of apoptosis decreased thereafter to normal levels.

In the present study, TUNEL assay was performed to assess the apoptosis of OT follicles. However, stromal cell apoptosis was not observed, as we thought that the outcomes of the follicle histological examination and apoptosis assay could reflect the stromal cell condition. Furthermore, we performed OT transplantation and analyzed the OT grafts on D2, D7, D21, and D42. These results would represent the quality of both follicles and stromal cells because if stromal cells were destroyed, the follicles could not survive or develop normally. Nevertheless, the lack of a direct analysis of stromal cell apoptosis could be a limitation of our study, and analysis of apoptosis both in OT follicles and stromal cells is desirable.

### Conclusions

In conclusion, our results showed that Ang2 treatment significantly improved angiogenesis after OT transplantation and resulted in an increase in the intact follicle ratios of the OT grafts. To our knowledge, this is the first study that used Ang2 for mouse OT transplantation, and we suggest that Ang2 treatment as an accelerator of angiogenesis could increase the success rate of fertility preservation. However, further studies using human OT are required before clinical application, and the reliability of the systemic effects of Ang2 should be verified.

## Supporting Information

S1 Table The results of mean follicle numbers and intact (grade 1; G1) follicle ratios according to different follicle development stages. (DOCX)
