# Supplementary material for: Effects of Angiopoietin-2 on Transplanted Mouse Ovarian Tissue
Source: PLoS One. 2016 Nov 21;11(11):e0166782. doi: 10.1371/journal.pone.0166782 (PMC5117712; doi:10.1371/journal.pone.0166782)
Supplement: S1 Table — (DOCX) [file pone.0166782.s001.docx]

**S1 Table.** The results of mean follicle number and intact (grade 1; G1) follicle ratios according to different follicle development stages.

|  | D2 | | | D7 | | | D21 | | | D42 | | |
| --- | --- | --- | --- | --- | --- | --- | --- | --- | --- | --- | --- | --- |
| Groups | **Control** | **50 ng Ang2** | **500 ng Ang2** | **Control** | **50 ng Ang2** | **500 ng Ang2** | **Control** | **50 ng Ang2** | **500 ng Ang2** | **Control** | **50 ng Ang2** | **500n g Ang2** |
| No. ovary | 23 | 27 | 26 | 22 | 24 | 30 | 31 | 32 | 28 | 21 | 24 | 24 |
| No. total follicle | 156 | 189 | 210 | 187 | 197 | 275 | 249 | 369 | 329 | 72 | 94 | 110 |
| Mean. follicle  (± SED) | 6.8 (± 0.7) | 7.0 (± 0.8) | 8.1 (± 0.9) | 8.5 (± 1.0) | 8.2 (± 0.8) | 9.2 (± 0.7) | 8.0 (± 0.8) | 11.5 (± 1.3) | 11.8 (± 1.0) | 3.4 (± 0.5) | 3.9 (± 0.5) | 4.6 (± 0.5) |
| G1 follicle (%) |  | | | | | | | | | | | |
| Primordial | 13/45 (28.9)**^a^** | 35/62 (56.5)**^b^** | 45/78 (57.7)**^b^** | 23/46 (50.0)**^a^** | 28/48 (58.3)**^ab^** | 75/102 (73.5)**^b^** | 44/61 (72.1) | 74/100 (74.0) | 48/63 (76.2) | 2/6 (33.3) | 7/11 (63.6) | 6/7 (85.7) |
| Primary | 16/30 (53.3) | 19/35 (54.3) | 24/43 (55.8) | 30/53 (56.6)**^a^** | 48/67 (71.6)**^ab^** | 52/62 (83.9)**^b^** | 40/73 (54.8)**^a^** | 52/85 (61.2)**^ab^** | 70/98 (71.4)**^b^** | 13/19 (68.4)**^ab^** | 15/27 (55.6)**^a^** | 27/34 (79.4)**^b^** |
| Secondary | 22/81 (27.2)**^a^** | 41/91 (45.1)**^b^** | 40/87 (46.0)**^b^** | 23/68 (33.8)**^a^** | 34/65 (52.3)**^b^** | 47/81 (58.0)**^b^** | 62/78 (79.5) | 99/134 (73.9) | 76/111 (68.5) | 9/36 (25.0)**^a^** | 21/37 (56.8)**^b^** | 26/50 (52.0)**^b^** |
| Antral | 0/0 (0.0) | 1/1 (100.0) | 0/2 (0.0) | 15/20 (75.0) | 17/17 (100.0) | 27/30 (90.0) | 31/37 (83.8)**^ab^** | 45/50 (90.0)**^a^** | 52/57 (91.2)**^b^** | 6/11 (54.5)**^a^** | 18/19 (94.7)**^b^** | 17/19 (89.5)**^b^** |

Control, no treatment of Ang2; 50 ng Ang2, injection of 50 ng/kg Ang2; 500 ng Ang2, injection of 500 ng/kg Ang2; D2, D7, D21 and D42, 2, 7, 21 and 42 days after transplantation, respectively.

*Different superscript letters indicate statistically signiﬁcant differences (p < 0.05), and the superscripts were used for the groups with the same transplantation duration.
